# Novel Star-Shaped Viologens Containing Phenyl and Triphenylamine Moieties for Electrochromic Applications

**DOI:** 10.3390/molecules29092006

**Published:** 2024-04-26

**Authors:** Radosław Banasz, Monika Wałęsa-Chorab

**Affiliations:** Faculty of Chemistry, Adam Mickiewicz University in Poznań, Uniwersytetu Poznańskiego 8, 61-614 Poznań, Poland

**Keywords:** star-shaped molecules, viologens, electrochromic applications, phenyl and triphenylamine core

## Abstract

The two star-shaped viologens containing 1,3,5-substituted phenyl (**1**) and triphenylamine (**2**) central cores and *n*-hexyl chains were synthesized and characterized. Both compounds exhibited promising optoelectronic properties and underwent multiple oxidation/reduction processes resulting in various colors. Four possible redox states of tripyridium salt containing a phenyl or triphenylamine core can occur depending on the applied potentials. The wide color range, from colorless through blue, azure to green-gray, was observed during the electrochemical reduction of compound **1**. In the case of compound **2**, the color change observed during spectroelectrochemical measurements was from yellow to colorless during the cathodic process and from yellow to green during the anodic process. The observed color change for both viologens was reversible. The triphenylamine-cored viologen (**2**) also exhibited emission in visible range and solvatochromism. It also exhibited luminescence in the solid state when excited with a UV lamp. These studies provide insights into the design of advanced materials for applications in displays.

## 1. Introduction

In the rapidly advancing development of modern technologies, the search for novel materials with unique properties is becoming a priority in the field of materials science and engineering. The viologens are one of the groups of compounds that attract a lot of attention because of their promising optoelectronic properties [1,2,3]. Viologens are the quaternary pyridinium salts and they are related to 4,4′-bipyridyl. These compounds are increasingly being studied for many types of applications, such as electrochromic displays [4,5], molecular machines [6,7] or redox flow batteries [8,9]. Viologen units can also be incorporated into covalent organic polymers, as presented by Trabolsi et al. [10], and, obtained this way, a multifunctional material was used as a “magic” ink for the sensing of ammonia and the effective capture of iodine vapors.

As-prepared linear viologens are usually in the dication form, which can be transformed to a radical cation via a one-electron reduction process. A second one-electron reduction leads to the formation of a neutral form of the viologen. Switching between different redox states results in very distinct color changes and this is the reason why they are intensively studied in terms of their electrochromic applications [11]. The superiority of viologens over other electrochromic materials is due to the fact that they usually exhibit good coloration efficiency and color contrast, and high redox stability. The electron transfer between redox states is fast because the process causes little structural changes. The simplest viologen, methyl viologen, is known to be colorless in the dicationic form and its color changes to deep blue when reduced. The intense color is attributed to a change transfer between the two nitrogen atoms of a different formal valence state (+1 and 0) [12]. However, EPR studies show that the charge and spin of the viologen radical cation are delocalized over the entire bipyridyl core [13]. The redox properties of viologens can be easily modified by altering the nitrogen substituents, which can be different aliphatic or aromatic groups [4,14]. Since the change of aliphatic chains does not change the optical properties of viologens much [15], the attachment of the aromatic groups results in a high modulation of electrochromic properties [16,17]. Another way of tuning the optical properties of viologens is via the introduction of different cores between 4-pirydyl groups. For example, viologens containing thiophene groups between 4-pirydyl groups exhibited a color change from colorless to purple, [18] while the introduction of a bithiophene group resulted in a liquid-crystalline material exhibiting a color change from yellow to green [19]. Woodward et al. synthesized and characterized viologens containing thiazolo[5,4-d]-thiazole as a bridging group and found that the obtained material showed an emission of light with a wavelength corresponding to the blue color and a reversible color change from yellow to dark blue as a result of the electroreduction [20]. The thienoviologens showed thermofluorochromism, which involves the switching of the emitted light from red to green and from white to blue as a result of the temperature-induced variation in the supramolecular interactions in the self-assembled phases [21]. Described by Veltri et al., *N*-aryl-substituted viologens based on bis(4-alkylphenyl)bipyridinium cations were found to exhibit liquid-crystalline properties and colorless-to-green electrochromism [22]. It has also been shown that the introduction of cores gives the molecule a bent structure, e.g., an oxadiazole spacer, interrupts conjugation and hinders electrotrochromism due to the absence of resonance coupling between the N-redox centers [23]. Furthermore, the composites of viologens with different carbon materials can be made to improve their stability and electrochromic performance [24,25,26].

The introduction of a branched core instead of a linear one results in obtaining star-shaped viologens. 1,3,5-Phenyl and triphenylamine (TPA) groups are great units to form branched viologens. So far, TPA-based viologens containing different N-substituents, such as boric acid groups [27], sodium propionate [28] or methyl groups [29], were found to exhibit luminescent properties and have been investigated for cell imaging. The branched viologens have also been used as building blocks for the construction of metal-organic frameworks [30], 2D supramolecular organic frameworks [31] and electroactive chiral covalent-organic frameworks [32]. However, the viologens containing 1,3,5-phenyl and triphenylamine cores have not been investigated for electrochromic applications.

**Figure 1:**
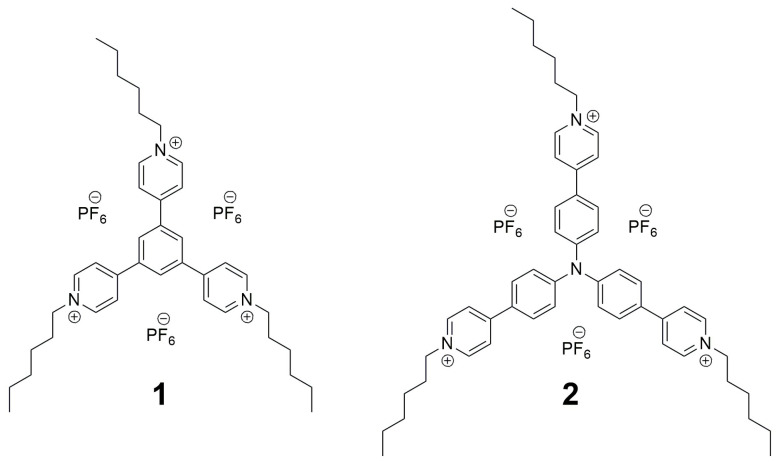
Chemical structures of the investigated viologens.

The star-shaped viologens have attracted a greater interest in recent years due to their clearly different properties compared to the linear viologens. Such compounds can exist in a higher number of redox states compared to the linear viologens and have become a unique alternative as electroactive materials. It is expected that the branched compounds containing three N-substituted pyridine rings will undergo more oxidation/reduction processes and thus exhibit more colors during electrochemical oxidation/reduction [33,34]. However, such viologens for electrochromic applications have not been widely investigated. In light of this we have decided to synthesize, characterize and investigate the electrochromic properties of two new star-shaped viologens, **1** and **2** (Figure 1), containing a 1,3,5-substituted phenyl group and triphenylamine. The compounds are N-substituted with *n*-hexyl chains. This was aimed at increasing the solubility of the compounds in organic solvents, as it is known that alkyl chain substitution is an effective chemical modification for improving the solubility of compounds [35,36,37]. This allowed for the investigation of the solvatochromic properties of the triphenylamine-based viologen (**2**).

## 2. Results and Discussion

### 2.1. Synthesis

**Figure 2:**
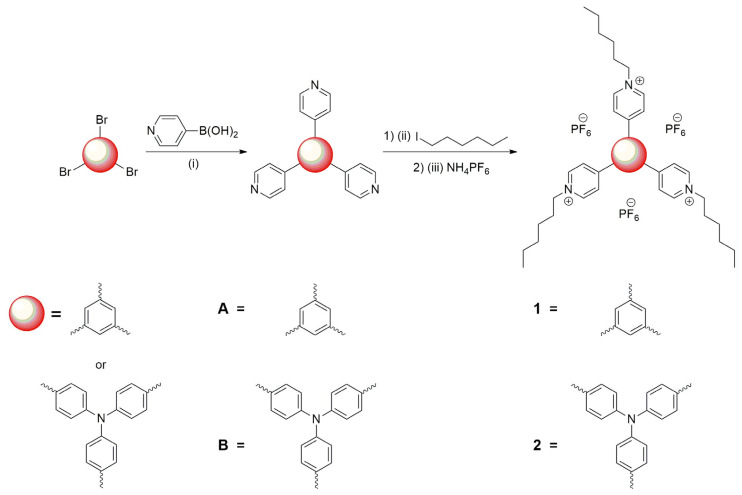
Synthetic scheme of the star-shaped viologens (**1** and **2**). Reaction conditions: (i) Pd(PPh_3_)_4_, K_2_CO_3_, THF/H_2_O, 60 °C, 72 h; (1) (ii) ACN/DMF, 140 °C, 1.5 h, microwave reactor; (2) (iii) NH_4_PF_6_, ACN/H_2_O, 25 °C, 24 h.

Phenyl- (**1**) and triphenylamine-based (**2**) star-shaped viologens containing hexyl groups were designed and prepared in a two-step synthesis (Figure 2).

Both the phenyl and triphenylamine are branching cores that can form star-shaped molecules for various electrochromic applications. Additionally, the triphenylamine group is known to have a three-dimensional structure that prevents stacking and enables luminescence [38]. In the first step, tripyridyl derivatives (**A** and **B**) have been obtained via a palladium-catalysed Suzuki-Miyaura coupling reaction between 1,3,5-tribromobenzene and tris(4-bromophenyl)amine cores, respectively, and 4-piridylboronic acid. The compounds have been obtained by modified protocols reported in the literature [39,40]. The bromination reaction to obtain (TPA)Br_3_ was conducted according to a previously described method [41]. In the next step, the microwave-assisted alkylation reaction between **A** or **B** and 1-iodohexane resulted in obtaining a targeted viologen in the form of iodide salts. The reactions were carried out in a dedicated microwave reactor in tightly closed, pressure-resistant vessels. The use of microwave-assisted synthetic methods allowed the acceleration of the synthesis and obtaining the compounds much faster, even in comparison to hydrothermal synthesis [42]. Further, to improve the solubility of the compounds in common organic solvents [43], the iodide salts have been converted to hexafluorophosphate salt via anion exchange. The solubility of **1** and **2** as PF_6_^−^ and I^−^ salts is shown in Table S1. It can be seen that the compounds as I^−^ salts were insoluble in ethyl acetate, THF and dichloromethane, while PF_6_^−^ salts could be solubilized in those solvents. The replacement of I^−^ anions with PF_6_^−^ was also done to exclude the oxidation/reduction peaks from the iodide anions, which could mask important signals from the reduction process of viologens in the voltammograms, since they are known to undergo an electrochemical reaction at low potentials [44,45]. The identity and purity of all compounds obtained have been confirmed by standard spectroscopic techniques; NMR spectroscopy and the HR-ESI-MS method (Figures S1–S15).

### 2.2. Electrochemical Properties

**Figure 3:**
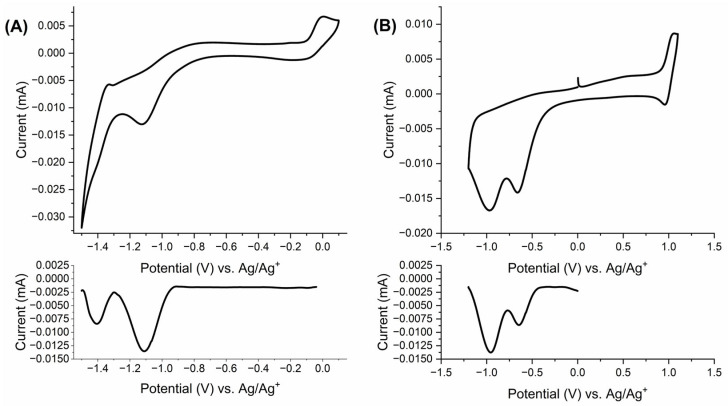
Cyclic voltammograms of star-shaped viologens (**A**) 1 and (**B**) 2 measured in an anhydrous and de-aerated 0.1 M solution of TBAPF_6_ in (**A**) DMSO, (**B**) ACN as a supporting electrolyte at 100 mV s^−1^. The corresponding differential pulse voltammograms are presented at the bottom of each panel.

To assess the usefulness of the star-shaped viologens **1** and **2** as electrochromic materials, their electrochemical properties were investigated. The electrochemical measurements were conducted via cyclic voltammetry at room temperature using a conventional three-electrode system containing a platinum disc working electrode, platinum wire as a counter electrode and an Ag/AgNO_3_ nonaqueous reference electrode. The cyclic voltammograms were taken at a 100 mVs^−1^ scan rate in a degassed and anhydrous 0.1 M solution of tetrabutylammonium hexafluorophosphate (TBAPF_6_) in DMSO for **1,** and acetonitrile for **2** as a supporting electrolyte. The cyclic voltammograms are shown in Figure 3.

**Figure 4:**
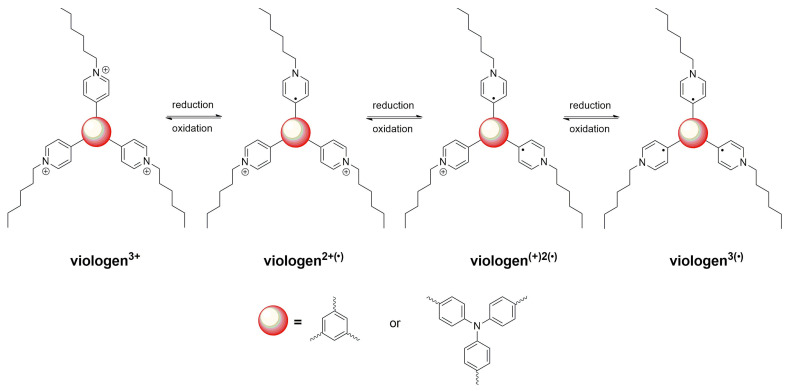
The mechanism of formation of different redox states in star-shaped viologens containing phenyl or triphenylamine groups.

Since three oxidation/reduction waves were expected for star-shaped viologens, for both **1** and **2** only two reduction waves in the cyclic voltammograms were found. In the case of **1**, the reduction waves have been observed at potentials E_pc_ = −1.12 V and E_pc_ = −1.39 V, while in the reverse scan, oxidation waves at E_pa_ = −1.33V, E_pa_ = −0.87 V and E_pa_ = −0.003 V were found (Figure 3A). This suggests that the two reduction processes [**1**^(3+)^]↔[**1**^(2+)(•)^] and [**1**^(2+)(•)^]↔[**1**^(+)(2•)^] overlap with a reduction potential of approximately −1.1 V. The same sequence of reduction processes was found to be better resolved in differential pulse voltammetry (DPV), wherein two differential current peaks appear at −1.1 and −1.4 V. The DPV peak at −1.1 V is broader and nearly twice as intense as the peak at −1.4 V, thus indicating poorly resolved first and second reduction events [46]. This was further supported by spectroelectrochemical measurements that allow the monitoring of the optical changes in situ during electrochemical process. The proposed mechanism of electrochemical reduction of the star-shaped viologens is shown in Figure 4. The four redox states of tripyridium salt containing a phenyl or triphenylamine core can occur depending on applied potentials. The as-prepared viologen is in the form of trication [viologen^(3+)^]. One-electron reduction leads to the [viologen^(3+)^]↔[viologen^(2+)(•)^] electrochemical process and formation of a radical dication. Next, two successive reduction processes lead to the formation of a diradical cation [viologen^(+)2(•)^] and finally a triradical cation [viologen^3(•)^].

In the case of **2**, an irreversible reduction potential was observed at E_pc_ = −0.66 V and a second quasi-reversible oxidation potential at E_pc_ = −0.97 V associated with the anodic wave at E_pa_ = −1.09 V (Figure 3B). Similar to **1**, the DPV of **2** contains two differential current peaks at −0.65 V and −0.95 V. The DPV peak at −0.95 V is broader and its intensity is higher than the peak at −0.65 V. This indicates that the redox process [**2**^(3+)^]↔[**2**^(2+)(•)^] occurs at −0.65 V, while the reductions [**2**^(2+)(•)^]]↔[**2**^(+)(2•)^] and [**2**^(+)(2•)^]]↔[**2**^(3•)^] occur at very similar potentials and are poorly resolved. Additionally, the reversible oxidation/reduction process with a half-wave potential of +1.0 V was observed, and was connected with the electrochemical process of the triphenylamine group and the formation of radical cation on triphenylamine [47,48,49,50].

### 2.3. Spectroelectrochemistry

**Figure 5:**
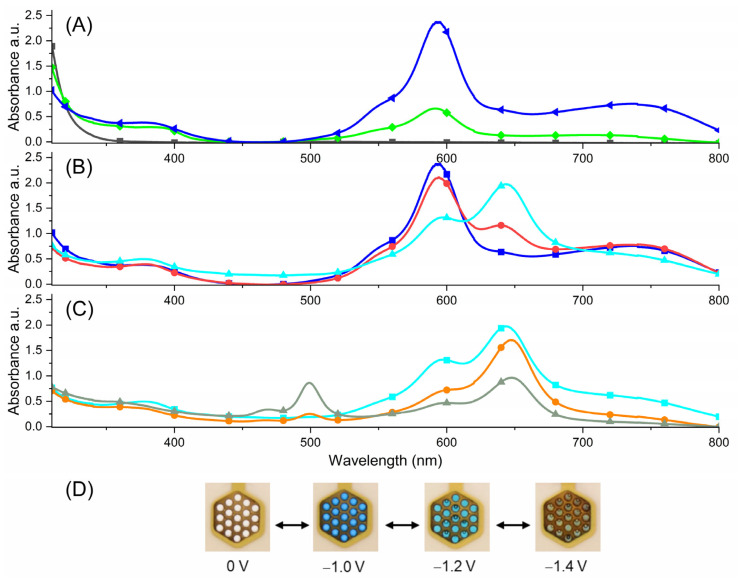
Spectroelectrochemistry of **1** measured in anhydrous and deaerated 0.1 M solution of TBAPF_6_ in DMSO as the supporting electrolyte, contingent on the applied potentials of (**A**) 0 V (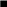) (black), −0.9 (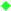) (green), −1.0 V (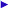) (blue); (**B**) −1.0 V (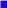) (blue), −1.1 V (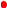) (red), −1.2 V (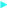) (cyan); (**C**) −1.2 V (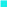) (cyan), −1.3 V (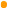) (orange), −1.4 V (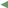) (grey-green); (**D**) photographs of the trication, radical dication, diradical cation and triradical (from left to right).

To further test the suitability of viologens in color switching devices, spectroelectrochemical measurements were performed. This was also done to determine the visible color changes during the electrochemical process. Spectroelectrochemical measurements were done using a commercially available honeycomb electrode. It was observed that in the neutral state, **1** is characterized by the absorption in the UV range below 300 nm and therefore it is colorless. When potentials up to −1.0 V were applied, the gradual formation of two bands in the visible region was observed with maxima at 594 nm and 735 nm (Figure 5A). This was accompanied by a perceived color change from colorless to blue and it was related to the formation of the radical dication form of the viologen [**1^(2+)(•)^**]. The clear isosbestic point at 320 nm confirms the presence of only trication and radical dication species in the solution and their independence. Further, applying a more negative potential in the range from −1.0 V to −1.2 V, a second color change from blue to azure was detected. This was the result of a decrease in the absorption intensity of the bands at 594 nm and 735 nm and the formation of a new absorption band at 643 nm (Figure 5B). The isosbestic point at 614 nm indicates that the radical dication is transformed into a diradical cation without any side products. This arises from the formation of the diradical cation of the viologen [**1^(+)2(•)^**]. The third redox process of **1** was assigned to the formation of the triradical [**1^3(•)^**] and was observed during a gradual decrease of applied potential in the range from −1.2 V to −1.4 V. This was visible as a color change from azure to green-gray and it was accompanied by a decrease in the intensity of the absorption band at 643 nm and formation of a new absorption band with a maximum at 499 nm (Figure 5C).

In comparison, the other star-shaped compounds, such as carbazole dibenzofurans [51], electrochemically polymerized star-shaped phenyl bithiophene [52], phosphaviologen oligomers with three or four phosphoryl-bridged viologen units [53], thiophene or EDOT-based oligomers with phenyl cores [54] or star-shaped thienoviologens [34], were found to exhibit only three color changes during the electrochemical process. In the case of viologens, a higher number of colors was observed for blend gel containing the mixture of ethyl viologen dibromide and 1,1′-bis(*p*-cyanophenyl)-4,4′-bipyridilium dichloride [55] or polymers bearing oligoaniline and viologen groups obtained through an electrochemistry-assisted hydrolytic crosslinking reaction [56]. The observed multicolor modulation of **1** during the electrochemical oxidation/reduction makes the compound an interesting multi-electrochromic material [57].

**Figure 6:**
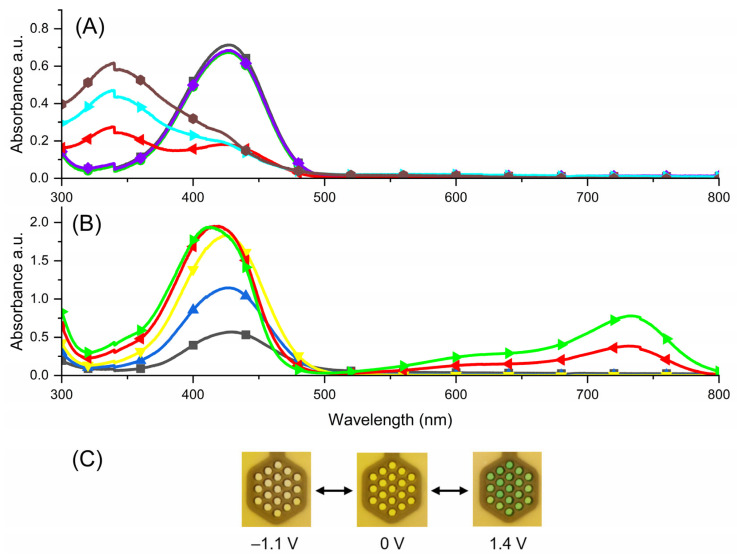
Spectroelectrochemistry of **2** measured in anhydrous and deaerated 0.1 M solution of TBAPF6 in acetonitrile as the supporting electrolyte contingent of applied potentials of (**A**) 0 V (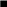) (black), −0.3 V (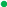) (green), −0.5 V (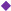) (violet), −0.9 V (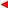) (red), −1.0 V (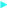) (cyan); −1.1 V (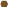) (brown) (**B**) 0.8 V (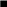) (black), 1.0 V (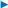) (blue), 1.1 V (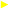) (yellow), 1.3 V (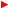) (red), 1.4 V (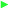) (green); (**C**) photographs of the compound in reduced state (left), trication form (middle) and oxidized state (right).

The spectroelectrochemical measurement for the viologen **2** with a TPA-core was done in a similar way as compound **1** (Figure 6). The trication form [**2**^(3+)^] was yellow and exhibited an absorption band in the visible range with a maxima at 427 nm. This was probably the result of the charge transfer process between the electron-accepting pyridium cation termini and the electron-donating triphenylamine core. After applying a negative voltage up to −1.0 V, only one color change was observed. The process of formation of radical dication, diradical cation and triradical was associated with the disappearance of the absorption band in the visible range and the formation of a new band in the UV range at 339 nm, which was perceived as a discoloration of the solution. This was reversible and when applying a slightly positive potential, a color change into yellow occurred. No isosbestic point was observed which indicates that different redox processes took place in the solution. During the oxidation process, the absorption band characteristic for the trication was restored and only a small shift of this absorption band (to 425 nm) was observed. This was concomitant with the formation of a new band at 732 nm, being the result of oxidation of the TPA-group [58,59]. A clear isosbestic point was observed at 520 nm. As a result, a color change from yellow to green was visible.

### 2.4. Luminescent Properties

Next, the luminescence properties in both solution in the solid state were investigated to determine the influence of the core on the emission properties of the compounds. Compound **1** was found to be non-emissive, while the viologen **2** containing triphenylamine was emissive in both solution and in the solid state. First, in order to assess the influence of solvent polarity on the optical properties of **2**, absorption and emission spectra were measured in solvents of different polarity: ethyl acetate, THF, dichloromethane, acetonitrile and methanol. Spectroscopic data for **2** are listed in Table 1.

**Figure 7:**
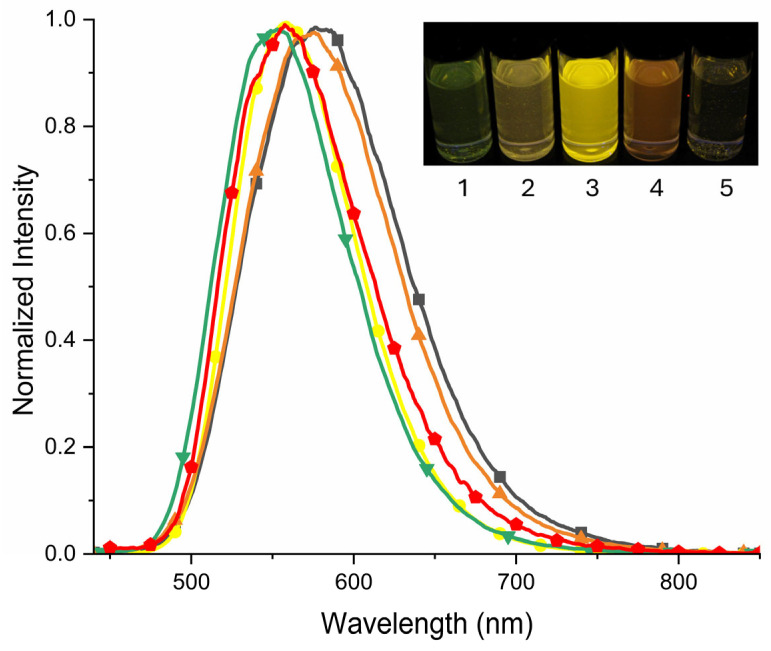
Normalized emission spectra of **2** in solvents of different polarities: ethyl acetate (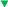) (green), tetrahydrofuran (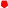) (red), dichloromethane (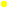) (yellow), methanol (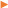) (orange) and acetonitrile (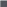) (black-grey). Insert: A photograph showing the emission of **2** in different solvents: 1—ethyl acetate, 2—tetrahydrofuran, 3—dichloromethane, 4—methanol and 5—acetonitrile, when excited with a UV lamp (λ = 365 nm).

The UV–Vis absorption spectra (Figure S16) exhibited an absorption band in the range between 427 nm to 450 nm. The emission maxima were found to be shifted from 550 nm in ethyl acetate to 577 nm in acetonitrile, correlating to an increase in the Stokes shift from 4966 cm^−1^ to 6088 cm^−1^. The increase in the Stokes shift was smaller compared to other triphenylamine donor-acceptor compounds [60,61] but it indicates that this compound in the excited state exhibits an ICT character. The emission of **2** involved a range of visible colors, including green (ethyl acetate), yellow (THF, dichloromethane and acetonitrile) and orange (methanol), when irradiated with the UV lamp (Figure 7). The fluorescence quantum yield (Φ_fl._) was also evaluated and it was observed that the fluorescence quantum yield of **2** was in the range of 4%–14%, depending on the solvent. One of the possible reasons for a low quantum yield might be the high conformational mobility of viologen [62]. As described in the literature, such conformational movement can be restricted upon intercalation into zeolites [63] or a polymer matrix [64,65], leading to increased emission. In the case of **2**, intramolecular movement is probably partially restricted by the incorporation of the bulky triphenylamine group.

**Figure 8:**
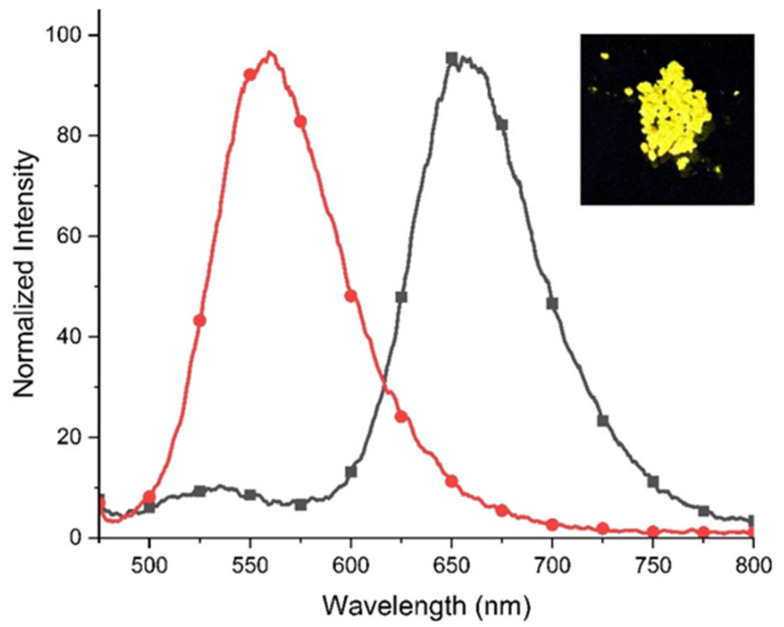
The excitation (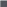) (black-grey) (λ_em_ = 657 nm) and emission spectra (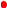) (red) (λ_ex_ = 560 nm) of **2** in the solid state. Insert: photo showing the emission of **2** in the solid state during excitation using a UV lamp (λ = 365 nm).

The excitation and emission spectra of **2** in the solid state are shown in Figure 8. As can be seen, viologen **2** in the solid state was also found to be luminescent. When excited at the excitation maxima (λ_ex_ = 560 nm), the powder exhibited emission in the visible range with a maximum at 656 nm and yellow emission was visible when irradiated with a handheld UV lamp (365 nm). The luminescence quantum yield of the solid was measured to be 5%.

## 3. Materials and Methods

### 3.1. General

A Bruker Avance 600 MHz apparatus was used in performing ^1^H NMR and ^13^C NMR spectral measurements. The analysis was performed in deuterated solvents from Deutero GmbH, and the spectra were referenced against the solvent signal (CDCl_3_ = 7.26 ppm, d_6_-DMSO = 2.5 ppm). A QTOF mass spectrometer (Impact HD, Bruker) in positive-ion mode was used as an instrument to measure HR-ESI-MS spectra.

Electrochemical measurements were performed using a Bio-Logic VSP potentiometer. The electrochemical measurements were conducted via cyclic voltammetry at room temperature, by using a conventional three-electrode system with a platinum disc working electrode, a platinum wire as a counter electrode and an Ag/AgNO_3_ nonaqueous reference electrode. The cyclic voltammograms were taken at a 100 mVs^−1^ scan rate in a degassed and anhydrous solvent (acetonitrile or DMSO) with 0.1 M tetrabutylammonium hexafluorophosphate (TBAPF_6_) as a supporting electrolyte.

Connecting a Bio-Logic VSP potentiostat (BioLogic, Seyssinet-Pariset, France) to a UV–Vis–NIR spectrometer (Jasco V770, Jasco, Tokyo, Japan) allowed us to conduct spectroelectrochemical measurements. The UV–Vis spectra and their color changes were measured by stepwise increasing the applied potential (the chronoamperometry method). The solution of viologens in solvents (acetonitrile or DMSO) with 0.1 M tetrabutylammonium hexafluorophosphate (TBAPF_6_) as the supporting electrolyte were tested using a honeycomb electrode (Pine Research Instrumentation, Inc., Durham, NC, USA) in a quartz cuvette with a silver wire as a reference electrode. To investigate the electrochromic behavior of the compounds, a solution of viologens with the supporting electrolyte was placed in a quartz cuvette with the gold honeycomb electrode as the working electrode, as well as a silver wire as the reference electrode. The visible color changes were recorded using a digital camera. Prior to the measurement, the silver wire was calibrated using ferrocene as an external standard [66,67] to make sure that the potentials during spectroelectrochemical measurements were consistent with those during the cyclic voltammograms.

Emission and excitation spectra were collected at room temperature in various organic solvents with a Jasco FP-8500 spectrometer (Jasco, Japan). The excitation and emission slits were 2.5 nm × 2.5 nm for solid-state measurements and 2.5 nm × 1 nm for studying solutions. The absorption of the solutions of the compounds for fluorescence measurements was around 0.1 at the excitation wavelength. The emission and excitation spectra of **2** in the solid state were measured using an integrating sphere (100 mm) which had been referenced using a calibrated light source. Absolute quantum yields were measured using an integrating sphere that had been calibrated using a calibrated light source.

The microwave-assisted reactions were conducted in a microwave reactor (Discovery 2.0 CEM Corporation).

### 3.2. 4,4′,4″-(Benzene-1,3,5-Triyl)Tris(1-Hexylpyridin-1-Ium) Tris(Hexafluorophosphate(V)) (***1***)

1-Iodohexane (0.24 mL, 1.62 mmol) was injected into a solution of compound A (0.1 g, 0.32 mmol) in an ACN/DMF mixture (5 mL, 10:1, *v*/*v*). The reaction mixture darkened during stirring and heating at 140 °C for 1.5 h in a microwave reactor. After cooling, the mixture was concentrated to the minimum amount of solution and then hexane was added to precipitate the viologen. The black residue was filtered off, washed with hexane and dried. The obtained powder was dissolved in an ACN/water mixture (20:1, *v*/*v*) and a solution of NH_4_PF_6_ (0.26 g, 1.60 mmol) in ACN was added. After the addition of salt, the reaction mixture was left stirring overnight at room temperature. The resulting dark brown mixture was extracted with dichloromethane and washed with water and brine. The separated organic phase was dried over magnesium sulphate and the solvent was evaporated under vacuum. The final product was obtained as a brownish solid (0.15 g, yield: 46%). ^1^H NMR (600 MHz, d_6_-DMSO) δ 9.34 (d, *J* = 7.2 Hz, 6H), 8.93 (s, 3H), 8.91 (d, *J* = 7.0 Hz, 6H), 4.67 (t, *J* = 7.5 Hz, 6H), 1.99 (q, *J* = 7.4 Hz, 6H), 1.37–1.29 (m, 18H), 0.88 (s, 9H) ppm. ^13^C NMR (100 MHz, d_6_-DMSO) δ 153.83, 153.44, 145.45, 140.18, 136.76, 133.21, 132.25, 130.80, 130.62, 128.93, 128.13, 116.41, 64.84 ppm. The HR-ESI-MS *m/z* = [**1** − (PF_6_^−^)_3_]^3+^ was calculated as 188.1434, and found as 188.1425 (20%), while the [**1** − (PF_6_^−^)_2_]^2+^ was calculated as 354.6974, and found to be 354.6958 (20%).

### 3.3. 4,4′,4″-(Nitrilotris(Benzene-4,1-Diyl))Tris(1-Hexylpyridin-1-Ium) Tris(Hexafluorophosphate(V)) (***2***)

A similar procedure to **1** was used: 1-Iodohexane (0.12 mL, 0.84 mmol) was injected into a solution of compound B (80 mg, 0.17 mmol) in an ACN/DMF mixture (5 mL, 10:1, *v*/*v*). The reaction mixture darkened during stirring and heating at 140 °C for 1.5 h in a microwave reactor. After cooling, the mixture was concentrated to the minimum amount of solution and then hexane was added to precipitate the viologen. The residue was filtered off, washed with hexane and dried. The obtained powder was dissolved in an ACN/water mixture (20:1, *v*/*v*) and a solution of NH_4_PF_6_ (0.14 g, 0.86 mmol) in ACN was added. After the addition of salt, the reaction mixture was left stirring overnight at room temperature. The resulting dark yellow mixture was extracted with dichloromethane and washed with water and brine. The separated organic phase was dried over magnesium sulphate and the solvent was evaporated under vacuum. The final product was obtained as an orange solid (0.15 g, yield: 46%).^1^H NMR (600 MHz, d_6_-DMSO) δ 9.08 (d, *J* = 7.3 Hz, 6H), 8.51 (d, *J* = 7.2 Hz, 6H), 8.17 (d, *J* = 9.1 Hz, 6H), 7.39–7.29 (d, 6H), 4.57 (t, *J* = 7.5 Hz, 6H), 1.98–1.91 (m, 6H), 1.30 (q, *J* = 4.1 Hz, 18H), 0.86 (s, 9H) ppm. ^13^C NMR (100 MHz, d_6_-DMSO) δ 153.36, 148.98, 144.55, 130.00, 128.81, 124.88, 123.68, 59.72, 40.06, 30.56, 25.07, 21.85, 13.81 ppm. The HR-ESI-MS *m/z* = [**2** − (PF_6_^−^)_3_]^3+^ was calculated as 243.8345, and found as 243.8349 (100%), while [**2** − (PF_6_^−^)_2_]^2+^ was calculated as 438.2342, and found to be 438.2339 (3%).

### 3.4. Tris(4-Bromophenyl)Amine (TPA)Br_3_

Triphenylamine (1.5 g, 6.1 mmol) was solubilized in 11.5 mL of CHCl_3_ and cooled down to 0 °C before the dropwise addition of bromine (0.98 mL, 18.3 mmol). The reaction flask was protected from light by using aluminium foil. After the complete addition of bromine, the green reaction mixture was stirred for 1.5 h. The resulting solution was added to distilled water and then was extracted with chloroform. The separated organic phase was dried over magnesium sulphate and the solvent was evaporated under vacuum. The crude yellow product was purified by recrystallisation in ethanol to obtain a final product as a white solid (2.62 g, yield: 89%). ^1^H NMR (600 MHz, CDCl_3_) δ 7.35 (d, *J* = 8.9 Hz, 6H), 6.92 (d, *J* = 8.9 Hz, 6H) ppm. ^13^C NMR (100 MHz, CDCl_3_) δ 145.99, 132.46, 125.55, 116.00 ppm. The HR-ESI-MS *m/z* = [(TPA)Br_3_+H]^+^ was calculated as 481.8572 and found to be 481.8579 (12%).

### 3.5. 1,3,5-Tri(Pyridine-4-yl)Benzene (***A***)

1,3,5-tribromobenzen (1.0 g, 3.18 mmol), 4-pyridylboronic acid (2.15 g, 17.49 mmol) and potassium carbonate (3.51 g, 25.40 mmol) were solubilized in a THF/water mixture (60 mL, 3/1, *v*/*v*) under an argon atmosphere. Tetrakis(triphenylphosphine)palladium(0) (0.09 g, 0.08 mmol) was added after degassing with argon for 15 min. The mixture was stirred and heated under reflux at 60 °C in an inert atmosphere for 72 h. After cooling, the reaction mixture was extracted with ethyl acetate and washed with water and brine. The separated organic phase was dried over magnesium sulphate and the solvent was evaporated under vacuum. The crude product was purified by column chromatography on silica gel using ethyl acetate/methanol/triethylamine (100/5/1) to give a product as a beige solid (0.61 g, yield: 62%). ^1^H NMR (600 MHz, CDCl_3_) δ 8.61 (d, *J* = 6.2 Hz, 6H), 7.88 (s, 3H), 7.59 (d, *J* = 6.2 Hz, 6H) ppm. ^13^C NMR (100 MHz, CDCl_3_) δ 150.64, 147.57, 140.52, 126.51, 121.99 ppm. The HR-ESI MS *m*/*z* = [**A +** H]^+^ was calculated as 310.1339, and found to be 310.1330 (100%).

### 3.6. Tris(4-(Pyridine-4-yl)Phenyl)Amine) (***B***)

A similar procedure to **A** was used: the compound (TPA)Br_3_ (0.40 g, 0.83 mmol), 4-pyridylboronic acid (0.57 g, 4.64 mmol) and potassium carbonate (0.92 g, 6.66 mmol) were solubilized in a THF/water mixture (40 mL, 3:1, *v*/*v*) under an argon atmosphere. Tetrakis(triphenylphosphine)palladium(0) (0.09 g, 0.08 mmol) was added after degassing with argon for 15 min. The mixture was stirred and heated under reflux at 60 °C in an inert atmosphere for 72 h. The separated organic phase was dried over magnesium sulphate and the solvent was evaporated under vacuum. The crude product was purified by column chromatography on silica gel using dichloromethane/acetone/methanol (100/10/1) to give a product as a pale yellow solid (0.23 g, yield: 58%).^1^H NMR (600 MHz, CDCl3) δ 8.65 (d, *J* = 6.2 Hz, 6H), 7.61 (d, *J* = 8.6 Hz, 6H), 7.50 (d, *J* = 6.2 Hz, 6H), 7.27 (d, *J* = 8.6 Hz, 6H) ppm. ^13^C NMR (100 MHz, CDCl_3_) δ 150.20, 147.77, 147.29, 132.80, 127.99, 124.61, 120.96 ppm. The HR-ESI-MS *m/z* = [**B +** H]^+^ was calculated as 477.2074, and found to be 477.2068 (100%).

## 4. Conclusions

In conclusion, the 1,3,5-substituted phenyl-based (**1**) and triphenylamine-based (**2**) star-shaped viologens containing hexyl groups were obtained and characterized by spectroscopic, electrochemical, spectroelectrochemical and luminescence methods. Due to a greater amount of N-substituted pyridine rings, compound **1** exhibited a higher number of oxidation/reduction processes compared to the viologens of a linear structure, which exhibited various colors during electrochemical oxidation/reduction. Viologen **1** was investigated, showing three reversible color changes, between colorless ↔ blue, blue ↔ azure and azure ↔ green-grey, which resulted in the formation of four different redox states. For viologen **2**, the reduction and oxidation process led to the reversible color changes, yellow ↔ colorless and yellow ↔ green, respectively. The luminescent properties of viologen **2** were investigated in the solid state and solution, demonstrating different emission colors depending on solvent polarity. The compound showed emission in the solid state under 365 nm UV lamp irradiation, thus it could be potentially applied as a light-emitting material. Both star-shaped viologens indicate promising optical properties and these molecules could be used as the active materials in electrochromic applications.

## Figures and Tables

**Table 1:** Spectroscopic data of **2** in various solvents.

Solvent	λ_abs_ [nm]	λ_em_ [nm]	Stokes Shift [cm^−1^]	QY [%]
Ethyl acetate	432 nm	550 nm	4966 cm^−1^	5%
THF	433 nm	557 nm	5141 cm^−1^	7%
Dichloromethane	450 nm	558 nm	4301 cm^−1^	14%
Acetonitrile	427 nm	577 nm	6088 cm^−1^	<1%
Methanol	429 nm	572 nm	5828 cm^−1^	4%

## Data Availability

The dataset generated during and/or analyzed during the current study are available from the corresponding author upon reasonable request.

## Supplementary Materials

The following supporting information can be downloaded at: https://www.mdpi.com/article/10.3390/molecules29092006/s1. Table S1: Solubility of **1** and **2** in different organic solvents. Figure S1: ^1^H NMR spectra of compound (TPA)Br_3_ in CDCl_3_; Figure S2: ^13^C NMR spectra of compound (TPA)Br_3_ in CDCl_3_; Figure S3: ^1^H NMR spectra of compound **A** in CDCl_3_; Figure S4: ^13^C NMR spectra of compound **A** in CDCl_3_; Figure S5: ^13^C NMR spectra of compound **B** in CDCl_3_; Figure S6: ^13^C NMR spectra of compound **B** in CDCl_3_; Figure S7: ^1^H NMR spectra of compound **1** in d_6_-DMSO; Figure S8: ^13^C NMR spectra of compound **1** in d_6_-DMSO; Figure S9: ^1^H NMR spectra of compound **2** in d_6_-DMSO; Figure S10: ^13^C NMR spectra of compound **2** in d_6_-DMSO; Figure S11: HR-ESI-MS spectra of compound (TPA)Br_3_; Figure S12: HR-ESI-MS spectra of compound **A**; Figure S13: HR-ESI-MS spectra of compound **B**; Figure S14: HR-ESI-MS spectra of compound **1**; Figure S15: HR-ESI-MS spectra of compound **2**; Figure S16: Normalized spectra of **2** in solvents of different polarities.
