# Supplementary material for: Novel Star-Shaped Viologens Containing Phenyl and Triphenylamine Moieties for Electrochromic Applications
Source: Molecules. 2024 Apr 26;29(9):2006. doi: 10.3390/molecules29092006 (PMC11085422; doi:10.3390/molecules29092006)
Supplement: Supplementary file 1 [file molecules-29-02006-s001.zip › molecules-2956943-supplementary.pdf]

SUPLEMENTARY INFORMATION

Novel star-shaped viologens containing phenyl and triphenylamine moieties for electrochromic applications

Radosław Banasz [1] and Monika Wałęsa-Chorab[1,*]

[1]*Faculty of Chemistry, Adam Mickiewicz University in Poznań, Uniwersytetu Poznańskiego 8, 61-614 Poznań, Poland*

Table of content

Table S1. Solubility of 1 and 2 in different organic solvents 2
Figure S1. ^{1}H NMR spectra of compound (TPA)Br_3 in $CDCl_3$ 3
Figure S2. ^{13}C NMR spectra of compound (TPA)Br_3 in $CDCl_3$ 3
Figure S3. ^{1}H NMR spectra of compound A in $CDCl_3$ 4
Figure S4. ^{13}C NMR spectra of compound A in $CDCl_3$ 4
Figure S5. ^{1}H NMR spectra of compound B in $CDCl_3$ 5
Figure S6. ^{13}C NMR spectra of compound B in $CDCl_3$ 5
Figure S7. ^{1}H NMR spectra of compound 1 in d_6-DMSO 6
Figure S8. ^{13}C NMR spectra of compound 1 in d_6-DMSO 6
Figure S9. ^{1}H NMR spectra of compound 2 in d_6-DMSO 7
Figure S10. ^{13}C NMR spectra of compound 2 in d_6-DMSO 7
Figure S11. HR-ESI-MS spectra of compound (TPA)Br_3 8
Figure S12. HR-ESI-MS spectra of compound A 8
Figure S13. HR-ESI-MS spectra of compound B 9
Figure S14. HR-ESI-MS spectra of compound 1 9
Figure S15. HR-ESI-MS spectra of compound 2 10
Figure S16. Normalized spectra of 2 in solvents of different polarities: ethyl acetate (▲ green), tetrahydrofuran (▼ red), dichloromethane (♦ yellow), methanol (● orange), acetonitrile (■ black) 10

Table S1. Solubility of **1** and **2** in different organic solvents

Solvent	**1** $PF6^-$	**1** I^-	**2** $PF6^-$	**2** I^-	
Ethyl acetate	+	-	+	-	
Tetrahydrofuran	+	-	+	-	
Dichloromethane	+	-	+	-	
Methanol	+	+	+	+	
Acetonitrile	+	+	+	+	

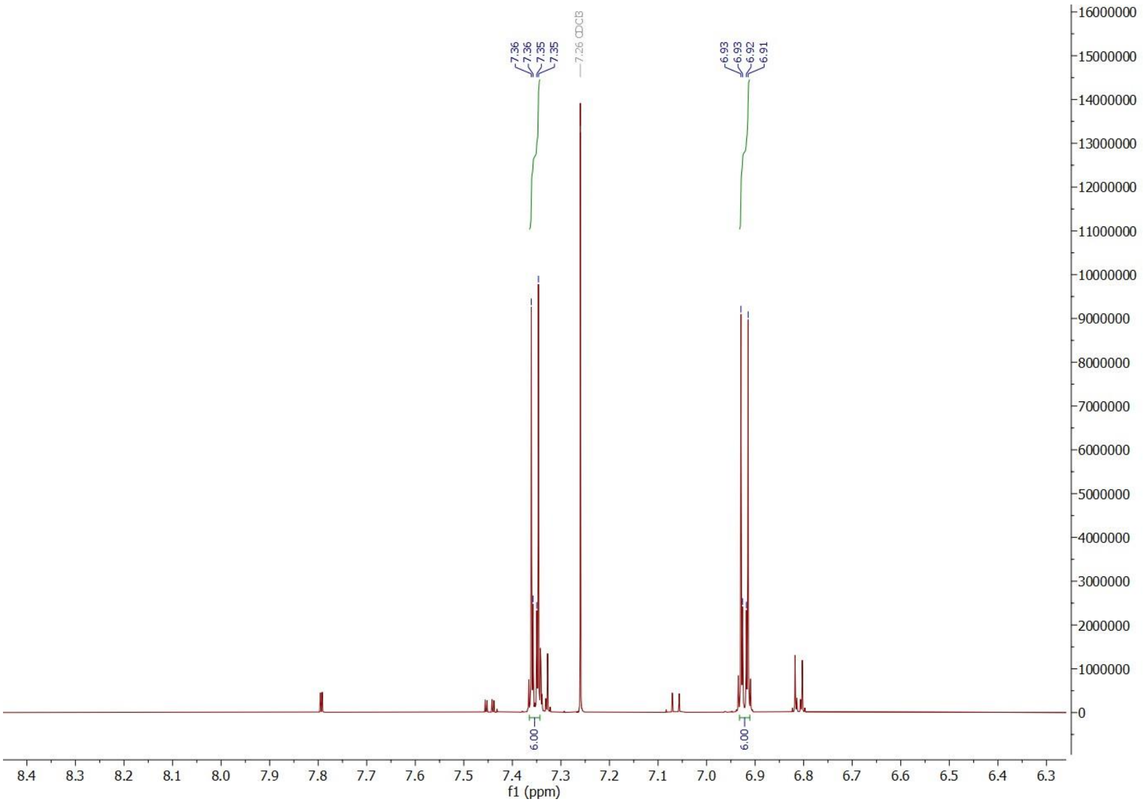

Figure S1. ^{1}H NMR spectra of compound **(TPA)Br₃** in $CDCl_3$

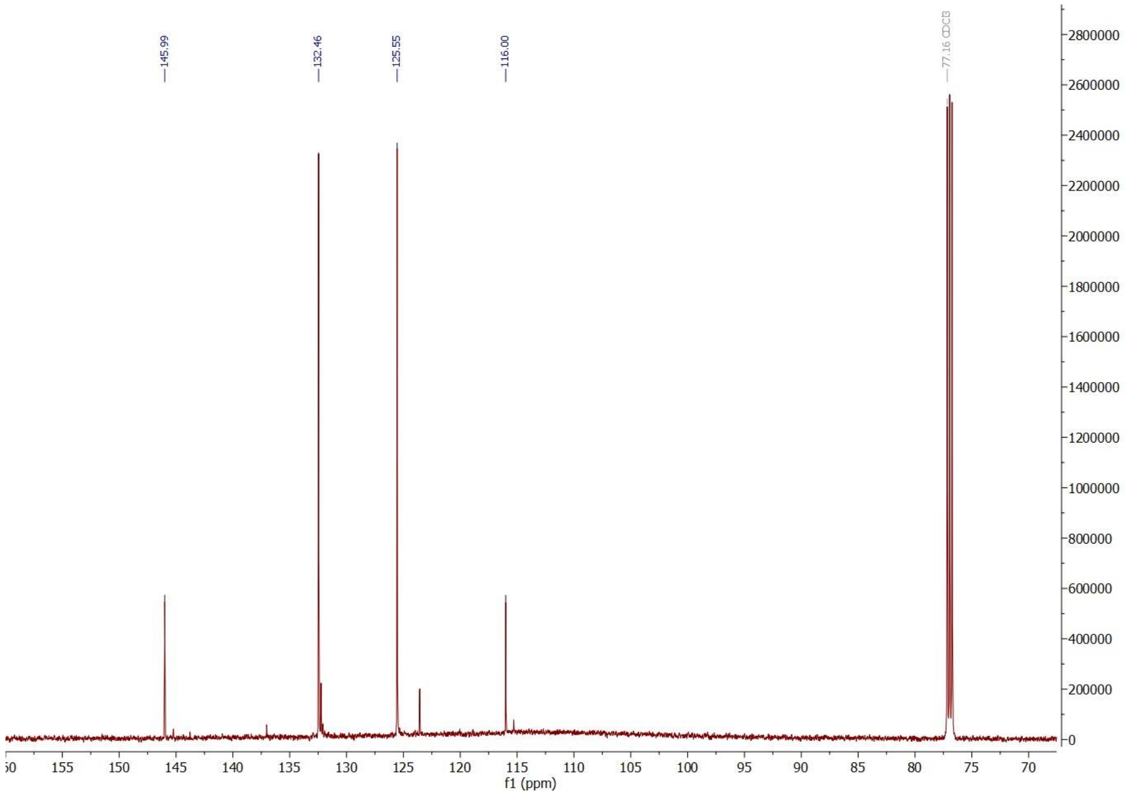

Figure S2. ^{13}C NMR spectra of compound **(TPA)Br$_3$** in $CDCl_3$

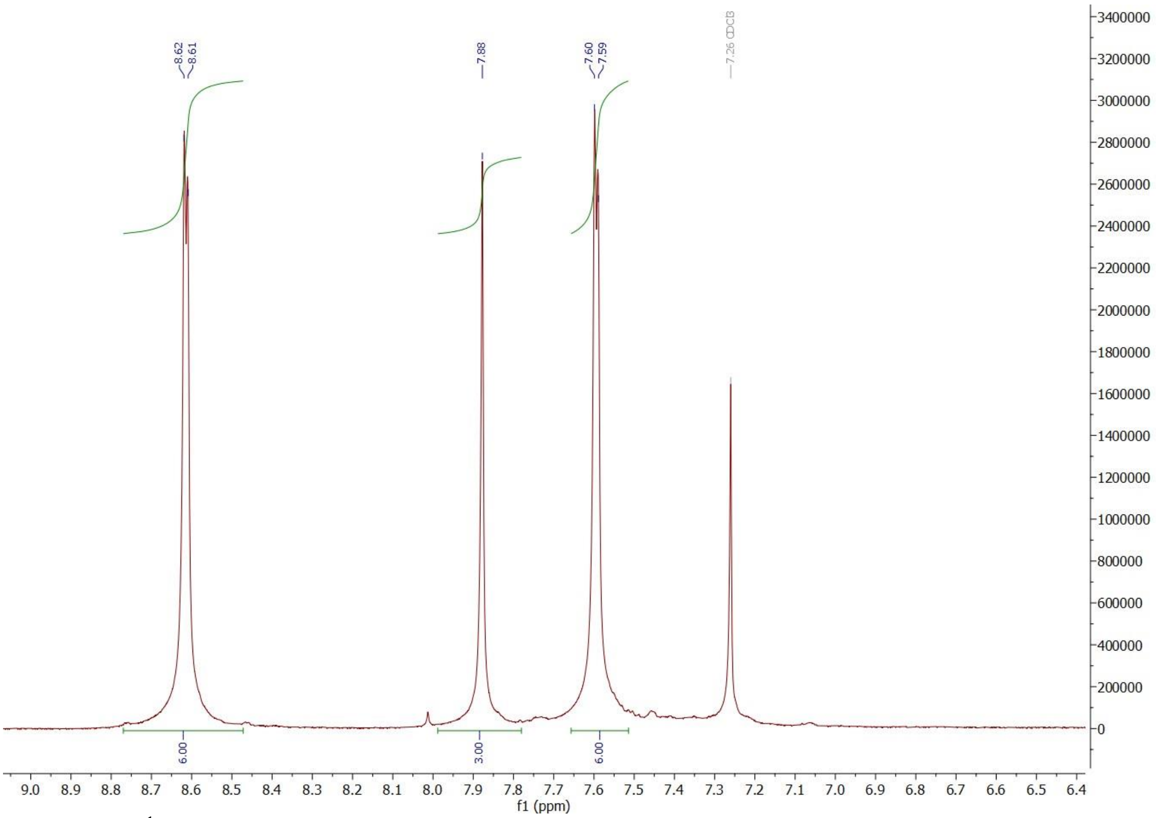

Figure S3. ^{1}H NMR spectra of compound **A** in $CDCl_3$

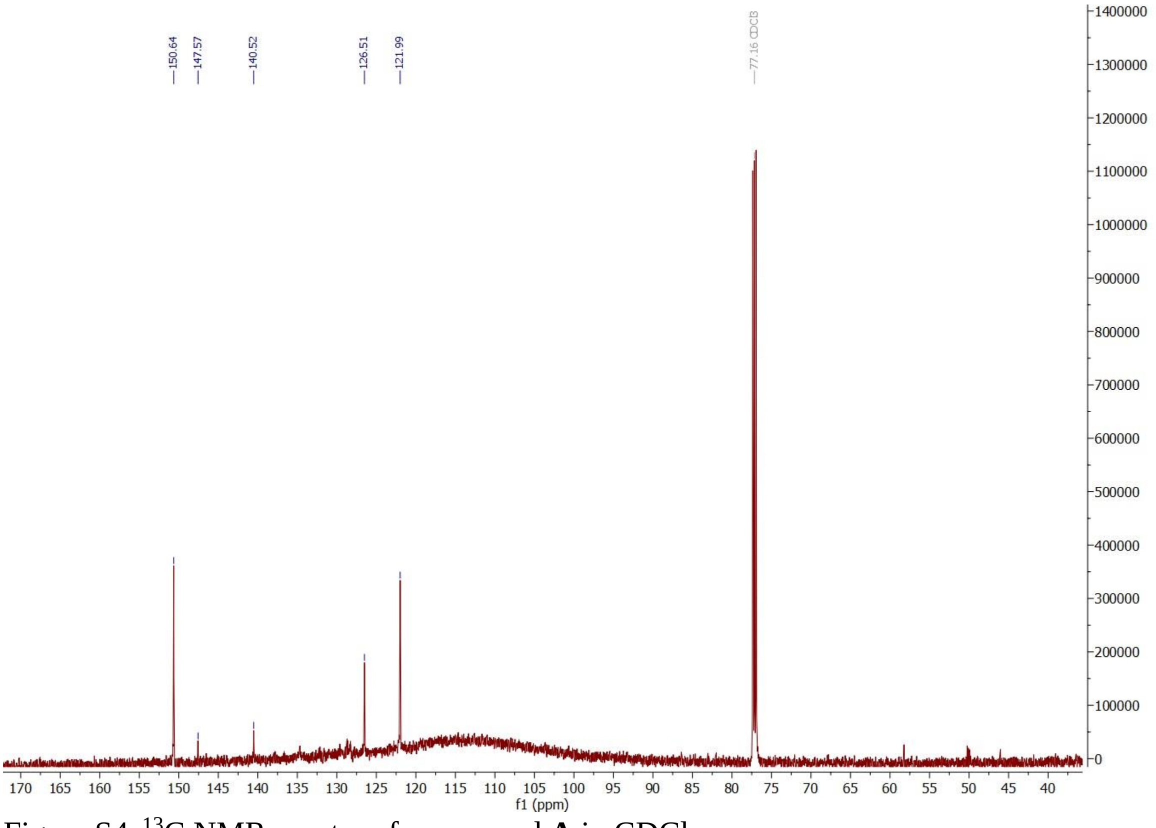

Figure S4. ^{13}C NMR spectra of compound **A** in $CDCl_3$

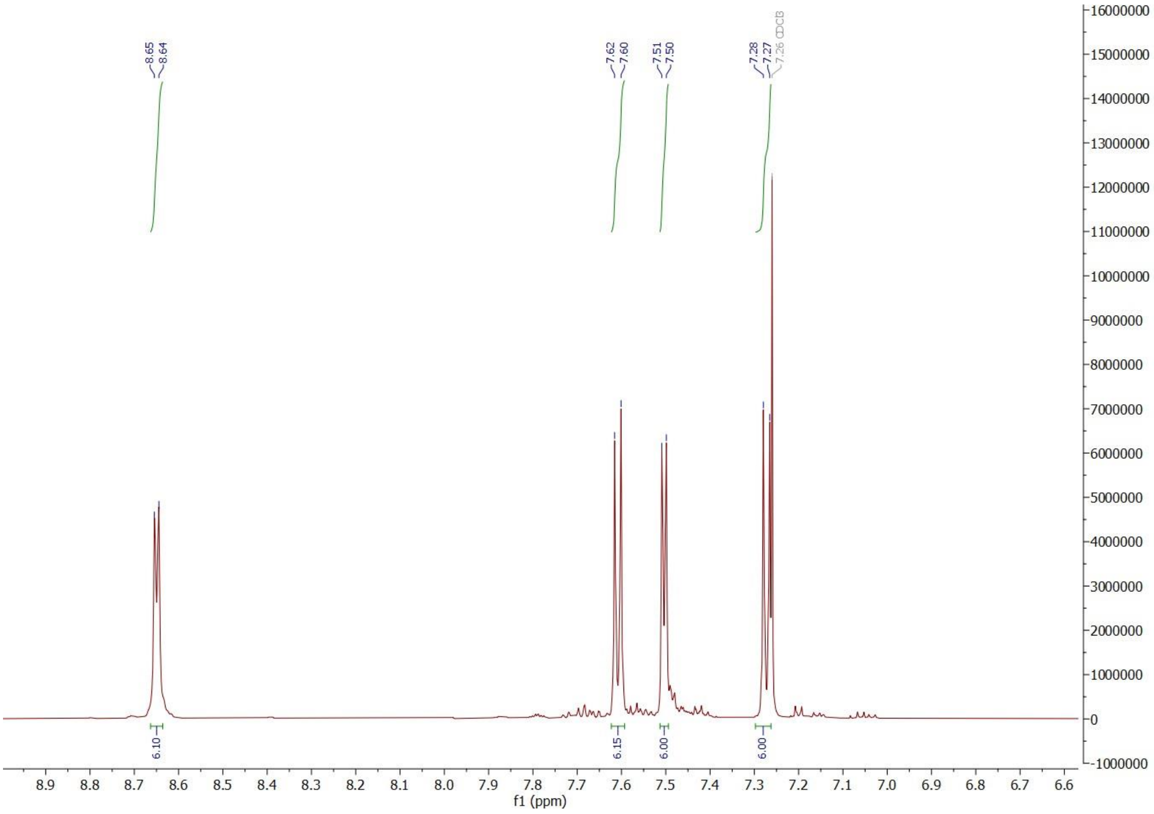

Figure S5. ^{1}H NMR spectra of compound **B** in $CDCl_3$

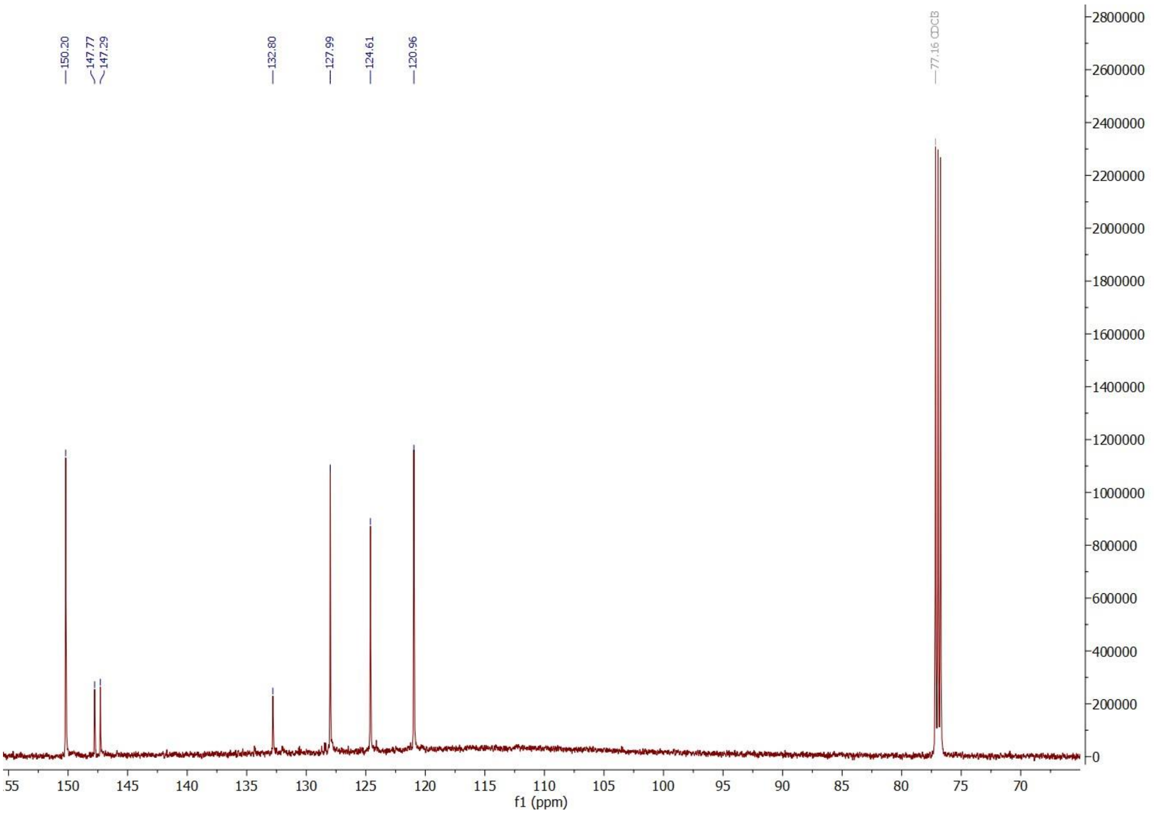

Figure S6. ^{13}C NMR spectra of compound **B** in $CDCl_3$

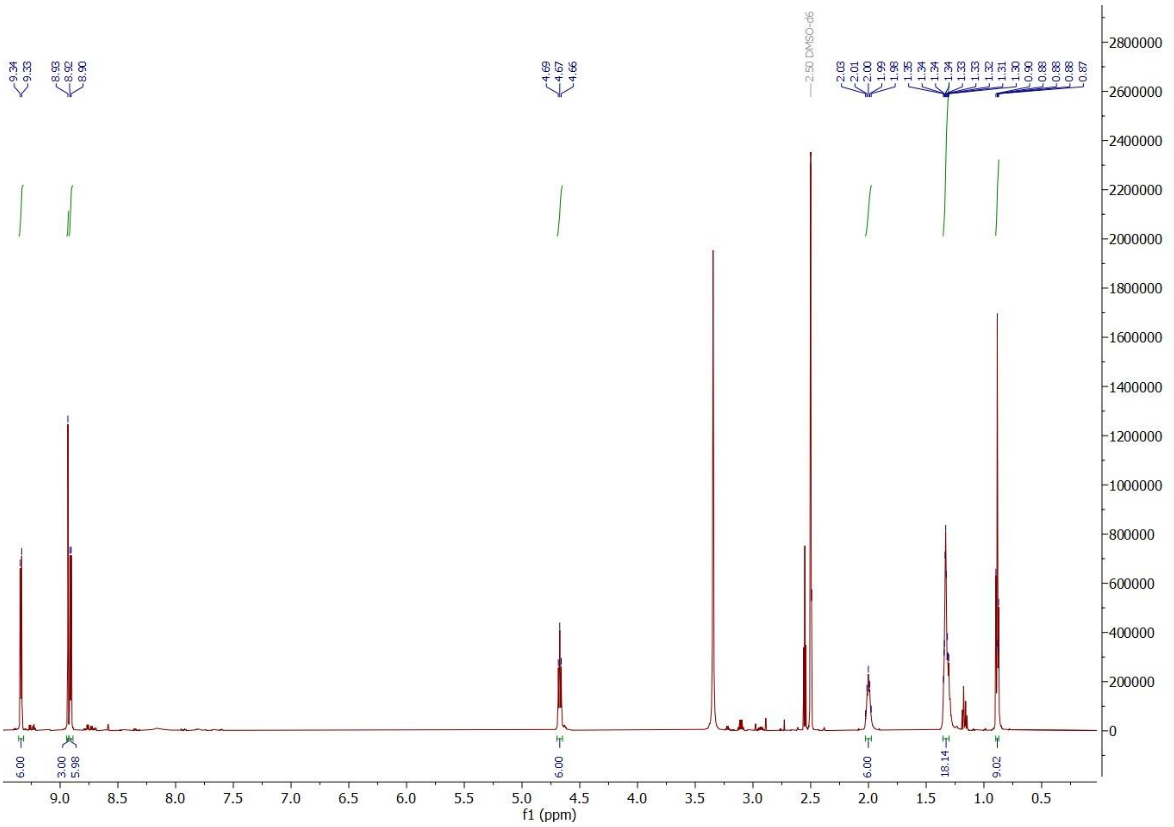

Figure S7. ^{1}H NMR spectra of compound **1** in d_6-DMSO

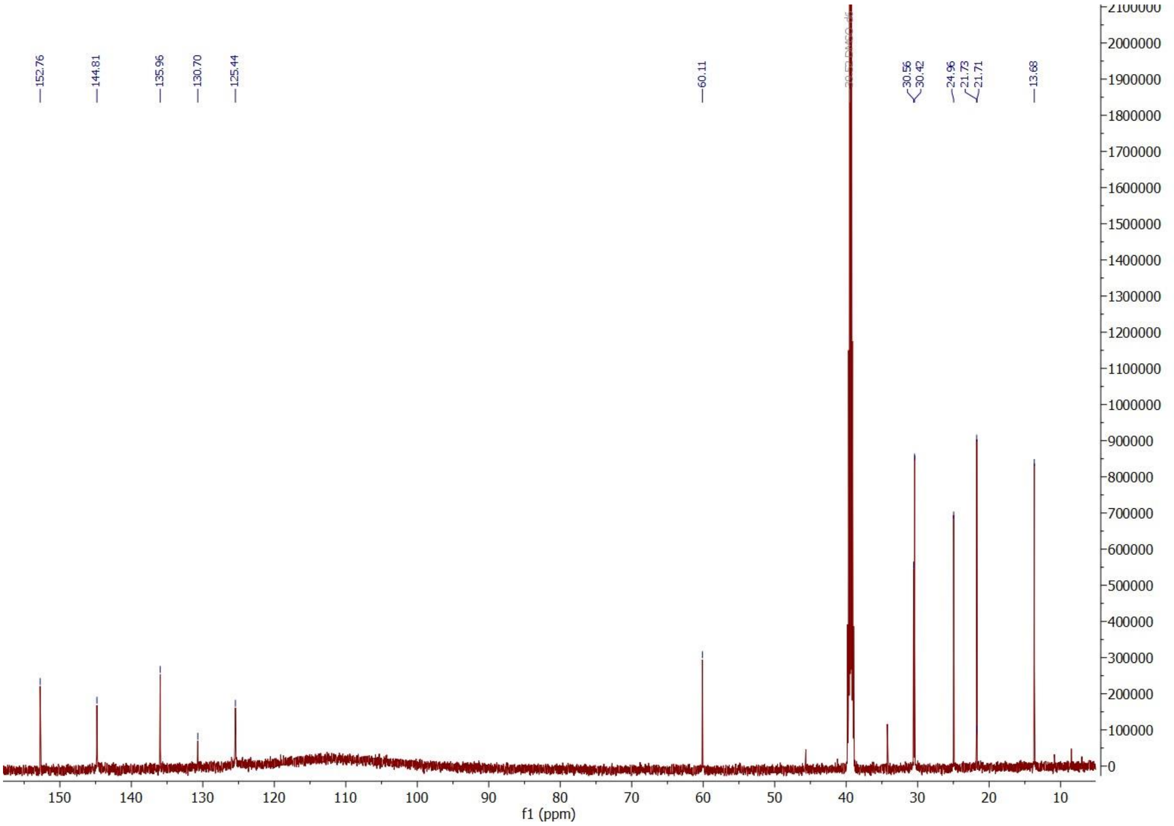

Figure S8. ^{13}C NMR spectra of compound **1** in d_6-DMSO

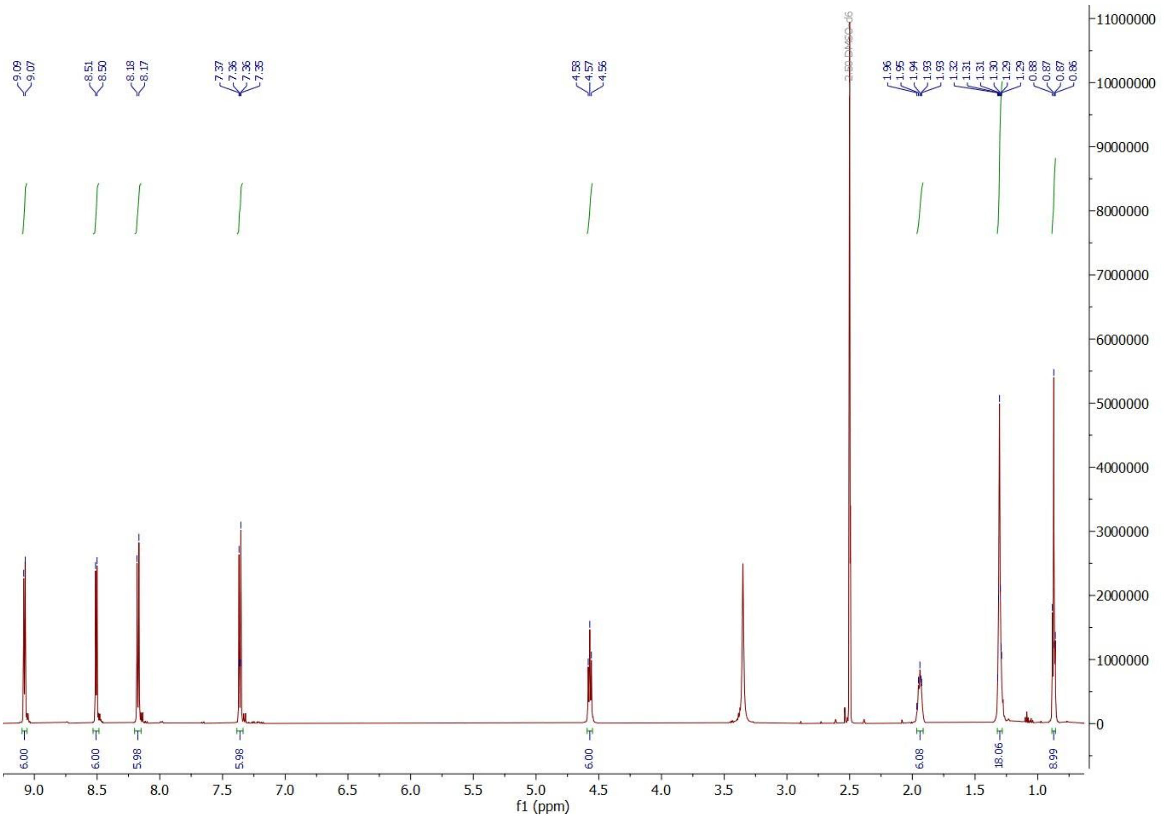

Figure S9. ^{1}H NMR spectra of compound **2** in d_6-DMSO

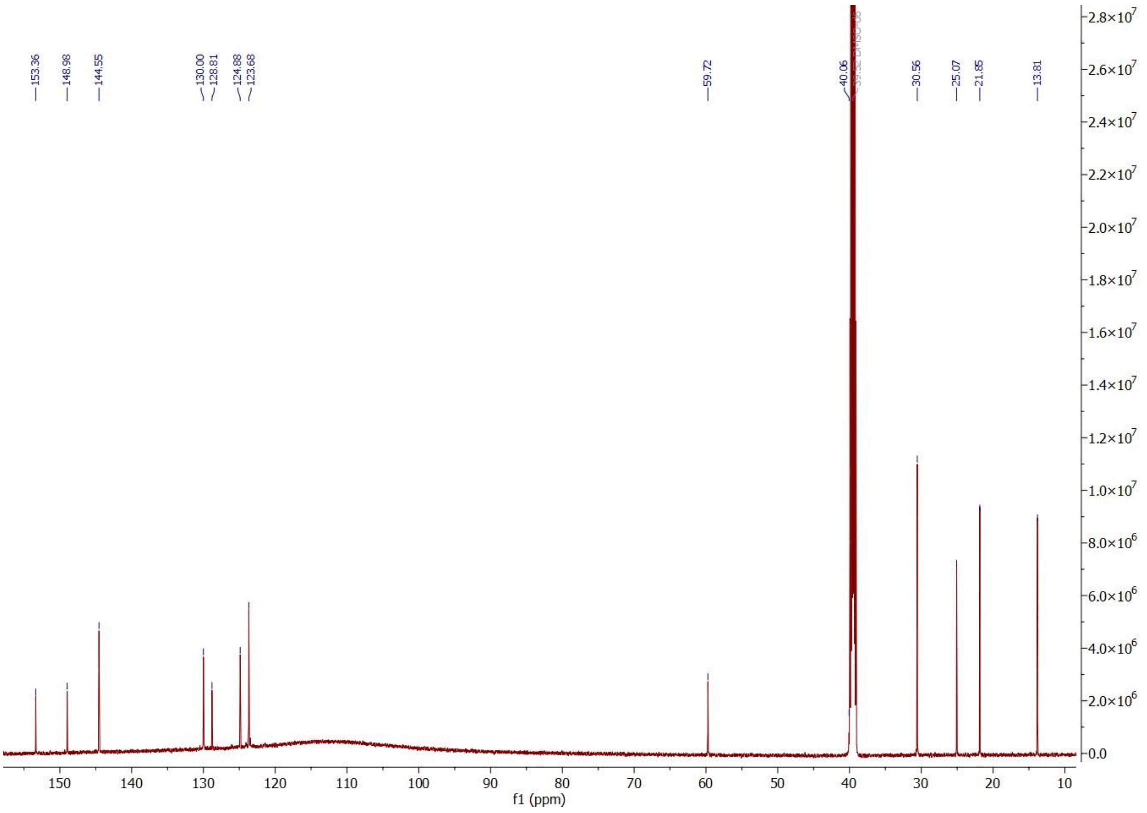

Figure S10. ^{13}C NMR spectra of compound **2** in d_6-DMSO

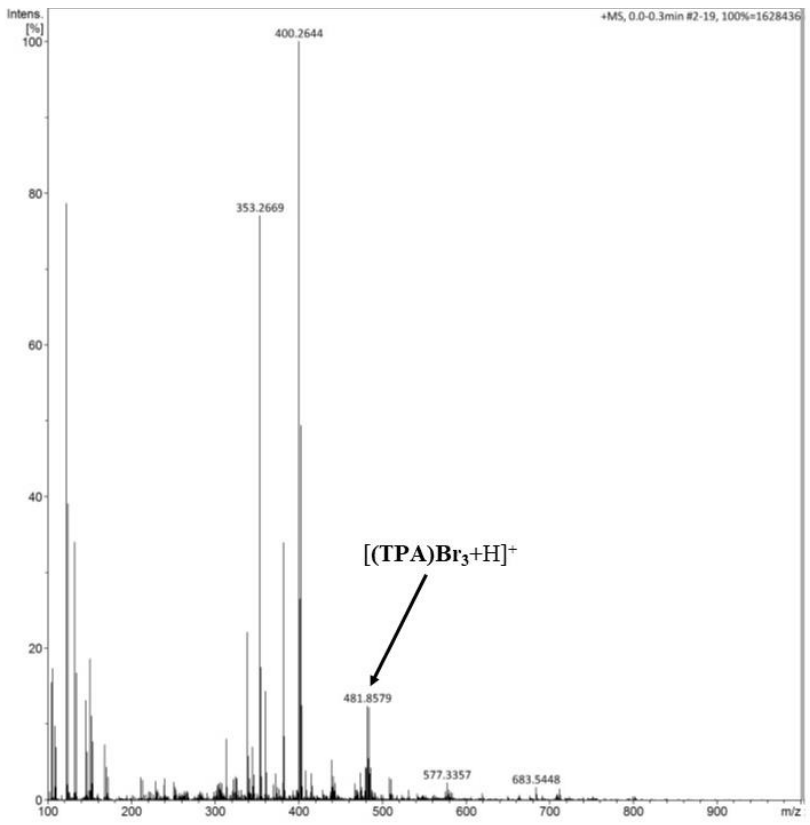

Figure S11. HR-ESI-MS spectra of compound **(TPA)Br$_3$**

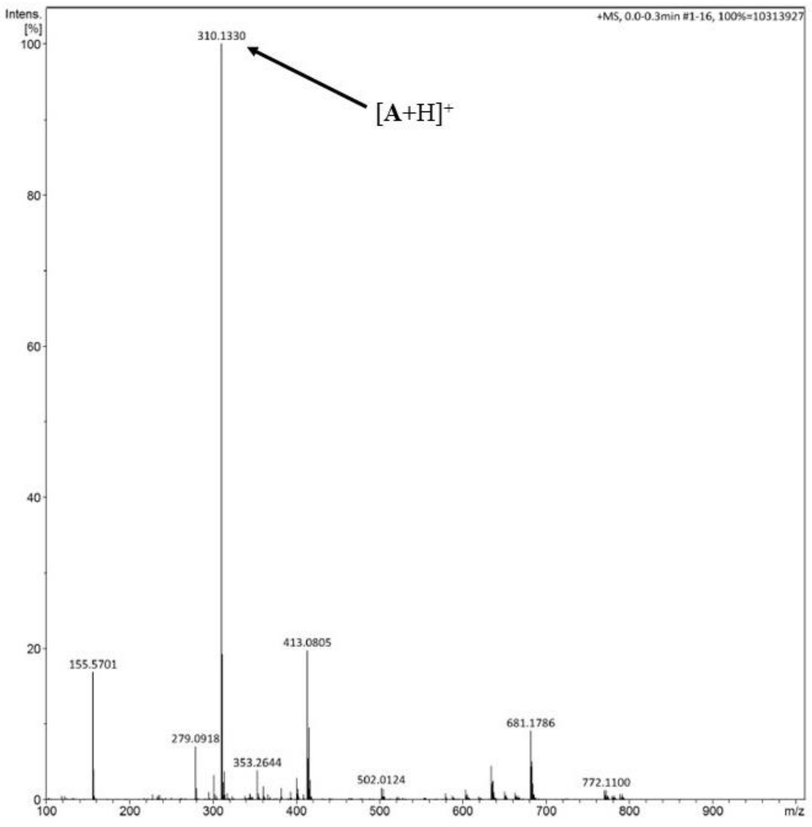

Figure S12. HR-ESI-MS spectra of compound **A**

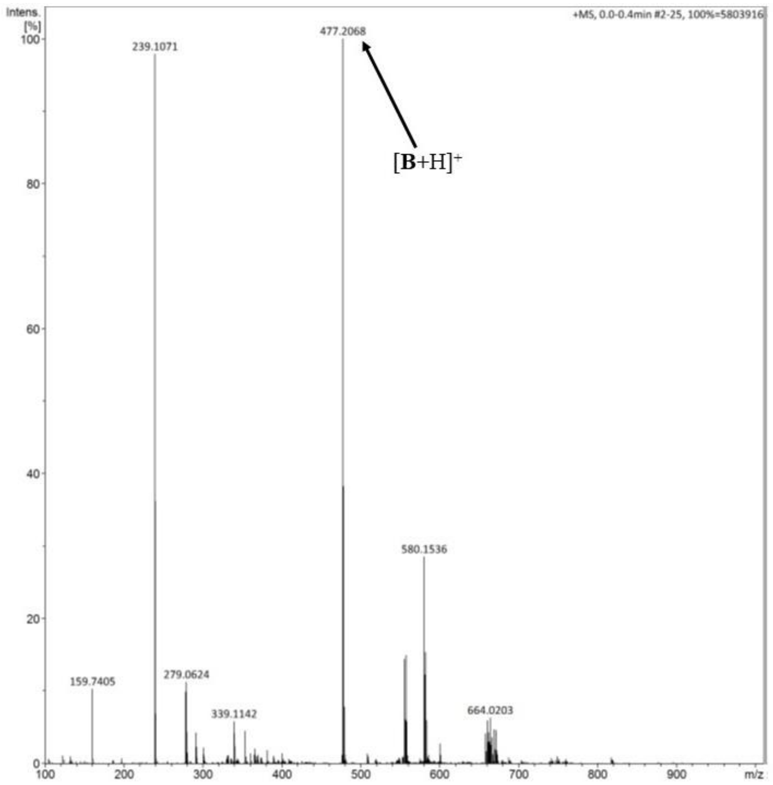

Figure S13. HR-ESI-MS spectra of compound **B**

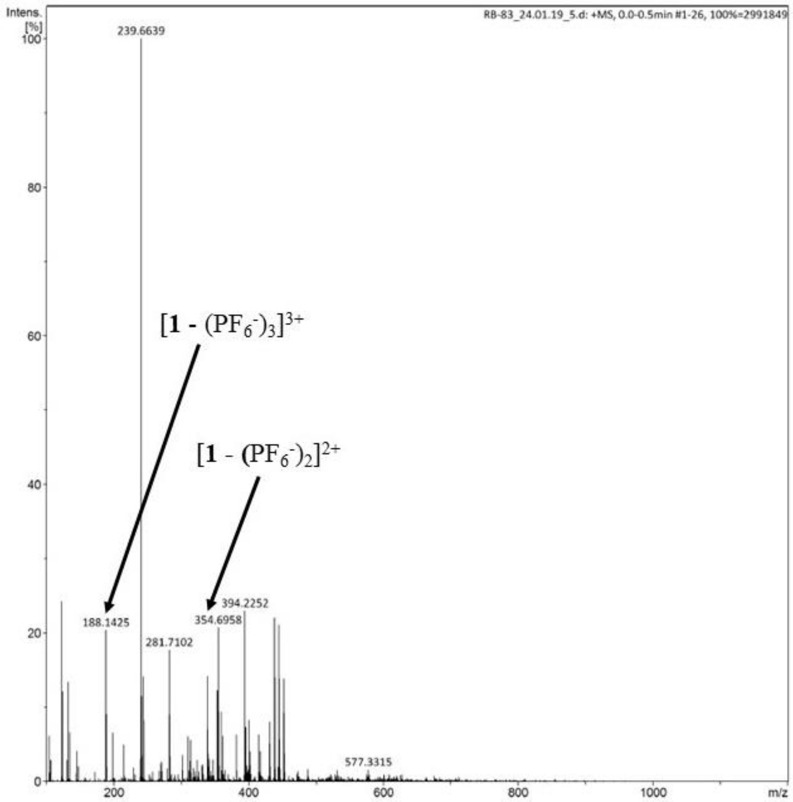

Figure S14. HR-ESI-MS spectra of compound **1**

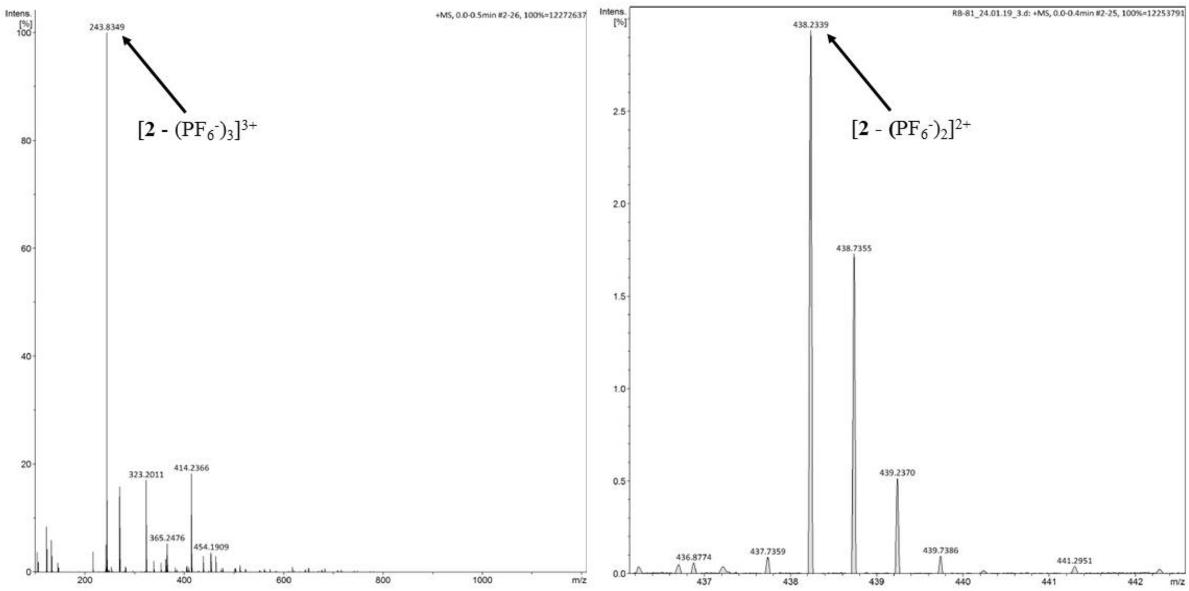

Figure S15. HR-ESI-MS spectra of compound **2**

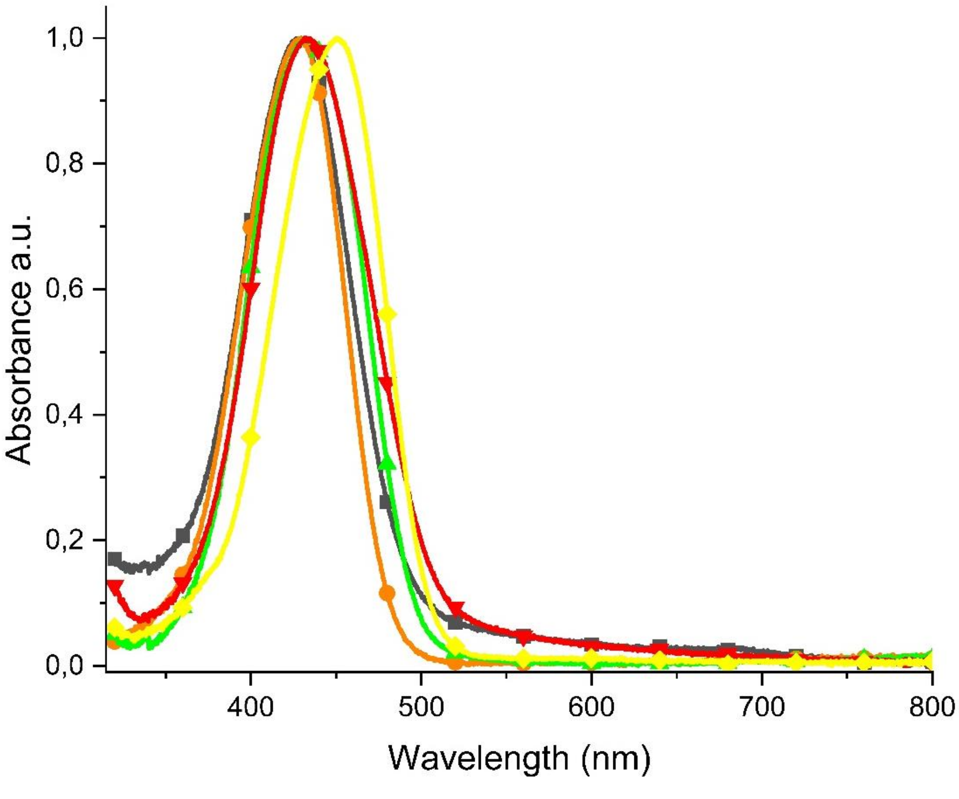

Figure S16. Normalized spectra of **2** in solvents of different polarities: ethyl acetate (▲ green), tetrahydrofuran (▼ red), dichloromethane (♦ yellow), methanol (● orange), acetonitrile (■ black).
